# Optimizing the Clinical Direction of Artificial Intelligence With Health Policy: A Narrative Review of the Literature

**DOI:** 10.7759/cureus.58400

**Published:** 2024-04-16

**Authors:** Mohit Lakkimsetti, Swati G Devella, Keval B Patel, Sarvani Dhandibhotla, Jasleen Kaur, Midhun Mathew, Janvi Kataria, Manisha Nallani, Umm E Farwa, Tirath Patel, Uzoamaka C Egbujo, Dakshin Meenashi Sundaram, Samar Kenawy, Mehak Roy, Saniyal Farheen Khan

**Affiliations:** 1 Internal Medicine, Mamata Medical College, Khammam, IND; 2 Medicine, Kempegowda Institute of Medical Sciences, Bangalore, IND; 3 Surgery, Narendra Modi Medical College, Ahmedabad, IND; 4 Gastroenterology, Massachusetts General Hospital, Boston, USA; 5 Emergency Medicine, Max Healthcare, Delhi, IND; 6 Internal Medicine, Trinitas Regional Medical Center, Elizabeth, USA; 7 Medicine, DY Patil University, Mumbai, IND; 8 Medicine, Kamineni Academy of Medical Sciences and Research Center, Hyderabad, IND; 9 Emergency Medicine, Jinnah Sindh Medical University, Karachi, PAK; 10 Medicine, American University of Antigua, Saint John's, ATG; 11 Pediatrics, Lagos University Teaching Hospital, Lagos, NGA; 12 Internal Medicine, Employees' State Insurance Corporation (ESIC) Medical College & Post Graduate Institute of Medical Science and Research (PGIMSR), Chennai, IND; 13 General Practice, Ministry of Health, Malé, MDV; 14 Internal Medicine, School of Medicine Science and Research, Delhi, IND; 15 Medicine, Governement General Hospital, Nizamabad, IND

**Keywords:** big data, computer vision, computer-aided detection, computer-aided diagnosis, convoluted neural network, deep neural network, ai and machine learning, deep learning artificial intelligence, emergency medicine - emergency critical care - disaster medicine

## Abstract

Artificial intelligence (AI) has the ability to completely transform the healthcare industry by enhancing diagnosis, treatment, and resource allocation. To ensure patient safety and equitable access to healthcare, it also presents ethical and practical issues that need to be carefully addressed. Its integration into healthcare is a crucial topic. To realize its full potential, however, the ethical issues around data privacy, prejudice, and transparency, as well as the practical difficulties posed by workforce adaptability and statutory frameworks, must be addressed. While there is growing knowledge about the advantages of AI in healthcare, there is a significant lack of knowledge about the moral and practical issues that come with its application, particularly in the setting of emergency and critical care. The majority of current research tends to concentrate on the benefits of AI, but thorough studies that investigate the potential disadvantages and ethical issues are scarce. The purpose of our article is to identify and examine the ethical and practical difficulties that arise when implementing AI in emergency medicine and critical care, to provide solutions to these issues, and to give suggestions to healthcare professionals and policymakers. In order to responsibly and successfully integrate AI in these important healthcare domains, policymakers and healthcare professionals must collaborate to create strong regulatory frameworks, safeguard data privacy, remove prejudice, and give healthcare workers the necessary training.

## Introduction and background

We have broken down the word cloud for artificial intelligence (AI) and its constituent terminologies as to what they stand for for better comprehension. Artificial intelligence is a multidisciplinary approach to computer science and linguistics that aspires to create machines capable of performing tasks that normally require human intelligence [1]. Artificial intelligence has been used increasingly in healthcare and has the capability to transform healthcare by improving risk prediction, supporting clinical decision-making, increasing the accuracy and timeliness of diagnosis, facilitating chart review and documentation, augmenting patient-physician relationships, and optimizing operations and resource allocation [2, 3]. Despite the advantages, abundant literature raises concerns about using AI in healthcare [4,5]. Aspects include privacy and consent, explainability of algorithms, workflow problems, and the frame problem, which is defined as unintended harmful effects from issues not related to patient care [6]. Machine learning (ML) is the process by which computer programs utilize data to identify patterns within the data and use these patterns to predict a relationship between the input and output [7]. Machine learning is the main component of AI, and there are three categories of ML algorithms: supervised learning, unsupervised learning, and reinforcement learning [8]. Deep learning (DL) is a popular ML technique that uses artificial neural networks (ANNs) to process input, passing it through many layers of interconnected nodes that resemble the functions of biological neurons. These neurons progressively detect data features and finally provide an output [7]. Artificial neural networks are computational models inspired by the structure and function of the brain's neural network that are used to perform computational problems. Similar to biological neurons, artificial neurons receive input from other neurons, are interconnected, and work collaboratively to process and analyze data. Artificial neural networks can be trained using supervised and unsupervised learning, and they can capture the complex relationship between input variables and outcomes and thus increase prediction accuracy [9]. 

Computer-aided diagnosis (CAD) refers to the application of ML in analyzing patient data and making assessments of patient conditions, which can then be used to assist healthcare professionals in making more accurate diagnostic decisions [10]. Natural language processing (NLP) is a field in AI that focuses on analyzing and processing text data, including written and spoken words. In simple terms, NLP allows computers to understand speech and text data and enables them to perform tasks such as language translation, speech recognition, text summarization, question answering, and many more [11]. Big data refers to large and complex data sets that exceed the scope of traditional data processing methods [12]. The characteristics of big data are five Vs namely, volume, velocity, variety, value, and veracity. Volume is the massive amount of data that is generated and collected from various sources, and these data have gone beyond traditional storage and analysis techniques. Velocity refers to the unprecedented speed at which data are generated and will require real-time processing to analyze and extract useful information from that data. Variety is the wide range of data types collected and includes structured, unstructured, or semi-structured data. Veracity refers to the data to reflect the truth or accuracy of the data. Value is the usefulness of data in providing information that drives informed decisions and actions [13-15].

Artificial intelligence applications in healthcare have changed the medical field, including imaging and electronic medical records (EMR), laboratory diagnosis and treatment, augmenting the intelligence of physicians, new drug discovery, providing preventive and precision medicine, biological extensive data analysis, speeding up processes, and data storage and access for health organizations. However, this field of science faces various ethical and legal challenges. Despite tremendous strides made in the field of AI in communities and its role in improving the treatment process, it is not accessible to all societies. Many low-income and developing countries still do not have access to the latest technologies. It should be noted that ethical dilemmas, privacy and data protection, informed consent, social gaps, medical consultation, empathy, and sympathy are various challenges that we face in using AI. Therefore, before integrating artificial intelligence with the healthcare system, practitioners and specialists should consider all four medical ethics principles, including autonomy, beneficence, non-maleficence, and justice in all aspects of healthcare [16].

## Review

Informed consent and autonomy

Informed consent is a procedure involving dialogue between a patient and a healthcare provider. It encompasses the assessment of the patient's ability to make decisions, the documentation of the informed consent process, and the ethical sharing of information [17]. In line with the concept of ethical obligation, patients possess the entitlement to receive information pertaining to their diagnoses, health conditions, treatment procedures, treatment outcomes, test findings, expenses, contributions from health insurance, or any other medical details. Any consent given should be precise in its intent, voluntary, and clearly understood. Concerns regarding this matter have also grown alongside the emergence of AI in healthcare applications [18]. Based on the autonomy principle, (a) every person has the entitlement to receive information and can pose inquiries prior to undergoing medical procedures and treatments; (b) patients should have the ability to comprehend the treatment procedures, potential screening and imaging risks, irregularities in data collection, programming errors, data privacy, access control, and the protection of a substantial amount of genetic information acquired from genetic testing; (c) patients have the right to decline treatment even if the healthcare provider believes it to be suitable; (d) patients possess the right to be informed about accountability in cases of failure or errors involving robotic medical devices. This information is crucial for both patient rights and the healthcare job market.

The philosophical foundation of autonomy, as understood by philosophers Immanuel Kant (1724-1804) and John Stuart Mill (1806-1873) and acknowledged as an ethical principle, is rooted in the belief that every individual possesses inherent and absolute value. Consequently, they should be empowered to make rational decisions and ethical judgments, with each person having the right to exercise their ability for self-determination [19]. In 1914, Justice Cardozo solidified this ethical principle in a court ruling with the concise statement, "Every mentally sound adult has the right to decide the fate of their own body"[20]. Autonomy, like all four ethical principles, must be carefully considered in relation to other moral principles, and there may be situations where it takes a backseat or is superseded. A clear example of this is when a patient's autonomous decision results in harm to other individuals. The principle of autonomy does not apply to individuals who do not possess the capability (competence) to make autonomous decisions. This includes infants, children, and individuals who lack competence due to developmental, mental, or physical illness. Protocols and policies have been set up by healthcare institutions and state governments in the US to evaluate the lack of competence. Nevertheless, a strict differentiation between the inability to make healthcare choices (evaluated by medical experts) and incompetence (established through legal proceedings) lacks practical utility. This is because a clinician's assessment of a patient's incapacity to make decisions due to physical or mental disorders carries identical practical implications as a legal verdict of incompetence [21]. Critics of the autonomy principle challenge its emphasis on the individual and advocate for a more encompassing idea known as relational autonomy. This perspective is influenced by social connections and intricate factors like gender, ethnicity, and culture [22]. Even within a highly developed Western nation like the United States, where the culture is diverse, certain minority communities possess distinct perspectives compared to the predominantly White population concerning the necessity of complete information sharing and choices regarding life-sustaining measures. These minority groups tend to favor an approach centered around family involvement [23].

It's not surprising to encounter opposition to the concept of patient autonomy and its related principles, like informed consent and truth-telling, in non-Western societies. In nations with deep-rooted traditions and ancient civilizations, the adoption of paternalism by physicians primarily stems from the principle of beneficence. Nevertheless, culture, which encompasses the collective beliefs, social norms, and material characteristics of a specific racial, religious, or social group, is not stagnant or self-contained; it evolves in conjunction with broader societal shifts over the years. It would be presumptive to assume that the established patterns and roles within physician-patient relationships, which have persisted for half a century or longer, remain applicable and unchanged. Hence, it becomes imperative to conduct a thorough assessment of medical practices characterized by paternalism. This is necessitated by various factors, such as advancements in technology and the economy, enhancements in the education and socioeconomic standing of the population, globalization, and the broader societal shift towards prioritizing the patient as an individual rather than merely a part of a collective. This essential inquiry can be achieved through research endeavors that encompass meticulously designed surveys covering demographic information and seeking insights into patient preferences regarding informed consent, truth-telling, and their involvement in the decision-making process. Honoring the autonomy principle imposes upon the healthcare provider the responsibility to divulge essential medical details and available treatment choices, thereby enabling the patient to assert their self-governance. This commitment underpins practices such as informed consent, transparent communication, and safeguarding confidentiality.

Confidentiality

Doctors have a duty to refrain from sharing confidential information provided by a patient with any third party unless the patient grants explicit authorization. A clear exception, implicitly authorized by the patient, involves the sharing of essential medical information from the primary physician to consultants and other healthcare teams for the purpose of patient care. In today's modern hospitals, characterized by numerous testing points, consultations, and the utilization of EMRs, there has been a gradual erosion of confidentiality. Nevertheless, individual physicians are required to exercise self-discipline by refraining from discussing patient-specific details with their family members, during social gatherings [24], or on social media platforms. There are a few significant exceptions to the rule of patient confidentiality. Included in these exceptions are legal mandates for reporting gunshot wounds and sexually transmitted diseases, as well as extraordinary circumstances that could result in significant harm to others, such as infectious disease outbreaks, notifying partners in cases of HIV infection, and informing relatives of specific genetic risks, among others.

The Health Insurance Portability and Accountability Act (HIPAA) was not written with AI in healthcare in mind; thus, applying old laws to new technology has rendered HIPAA privacy standards obsolete [25]. Current policies should have addressed the problems raised by AI developers who used enormous volumes of data to construct AI software, such as patient data and data mining. Thus, current regulations must be revised when technology advances [25]. The Health Insurance Portability and Accountability Act must be updated to keep up with current trends in innovation and technology without impeding their advancement. As AI in healthcare has become increasingly popular in medicine, data privacy experts have continued to raise concerns about the ethical implications [26]. The US Food and Drug Administration (FDA) has made its first attempt to create criteria for analyzing the safety and efficacy of AI systems [7]. The new General Data Protection Regulation (GDPR) of the European Union (EU) establishes a single system applicable to all personal data and its protection, which is far larger than HIPAA [27]. Similarly, the National Health Service (NHS) is developing standards for proving the efficacy of AI-driven technology to avoid complicating innovation and uptake throughout the screening process [7]. Both initiatives are ongoing and pose a hurdle to accepting AI-based interventions by courts and regulatory bodies. Adopting a separate regime for health-related data that is not covered by HIPAA is the best choice [27]. With standard rules for how AI should be utilized, it is clear how far AI can be ethically used in hospitals. 

Artificial intelligence has streamlined patient care by providing faster and more accurate diagnoses, analyzing data for improved treatments, and making healthcare providers more efficient and productive [27]. Healthcare workers have been concerned about how AI will manage patient data without violating HIPAA. Concerns about AI's potential influence on health data privacy have been raised by ML software and organizations that develop AI but are not subject to HIPAA rules [25]. The health information technology revolution has permitted the compilation and use of massive data sets of health records. However, the manipulation of large amounts of health information has presented issues ensuring the privacy of patients and research subjects [28]. The HIPAA allows the unrestricted exchange of de-identified health information for research and commercial purposes. However, AI techniques, such as ML algorithms, may piece together information to re-identify a person even if their pertinent identifying information has been removed [25]. Concerns about AI's potential influence on health data privacy have been raised by machine learning software and organizations that develop AI but are not subject to HIPAA rules. The HIPAA does not apply to health or healthcare data generated by non-covered companies, nor does it apply to patient-provided health information, such as social media posts [27]. It needs to address the massive amount of data that is not directly related to health but allows for conclusions about health.

Machine learning and deep learning models require large datasets. However, data availability in healthcare is a complicated topic [7]. There must be plans to securely and efficiently move electronic health records (EHR) data between healthcare providers to provide a higher-quality healthcare experience [29]. The massive amount of data necessary for deep learning raises obvious privacy concerns. Companies that collect users' personal, highly sensitive data keep it indefinitely. Users cannot delete it or limit the purposes for which it is utilized. Patient consent is thus an important component of data privacy concerns, as healthcare organizations may allow large-scale usage of patient data for AI training without securing appropriate individual patient approval [7]. Personal privacy must always be protected when clinical data is used for secondary purposes [30]. The right of patients to control secondary uses of their clinical data is a recurrent ethical dilemma. Data sharing is becoming more widely regarded as essential for cross-disciplinary research and scientific legitimacy. It requires a framework to determine what ethical norms should govern it [31]. While some urge individual data control, ethical standards do not support a patient's right to prevent or profit from knowledge acquired from de-identified data that poses no harm to the patient [30]. Others who favor the ability to exclusively license the data, on the other hand, believe that it is unethical for an organization to get access to and profit from actions generated by these insights while restricting others from gaining access to the same source of insights [30]. Individuals and corporations that have access to healthcare data must accept responsibility for safeguarding protected health information. For example, they must not employ exclusive licensing to prevent others from learning from the data [30]. Any parties involved must ensure that any data-derived information is used constructively. It is in the public's best interest to ensure that all users of this resource adhere to these ethical standards [30].

Clinical application of AI

Breast cancer is a devastating disease with a high global burden [32], for which early screening and diagnosis are imperative. A systematic review identified three retrospective studies that compared an AI system against a trained human radiologist’s clinical decisions in the screening for breast cancer. This review utilized a dataset of 79,910 women, which includes 1,878 women who had cancer detected or developed within 12 months of screening. The review concluded that 94% of the evaluated AI systems were less accurate than a single trained human radiologist, with all tested systems being less accurate than a group of two or more radiologists convening to make the clinical decision [33]. The authors of this review note that many of the studies examined are of poor methodological quality and have mentioned additional smaller studies with high risks for bias and low generalizability that report AI systems as more accurate than a laboratory radiologist, with the ability to operate as a standalone radiologist or reader aid [33]. The limitations of studies in this area of medicine are underscored by the authors, with a clear need for a validated and standardized protocol. A meta-analysis published in the Lancet details a comparison between 34 studies on the early diagnosis and detection of tumor metastasis, with a pooled sensitivity of 82% and specificity of 84%. Only studies examining tumor metastasis diagnosis by medical images (i.e., contrast ultrasound, PET/CT, MRI, etc.) were included, other methods of diagnosis, such as histopathology, were excluded. This is an important area of study due to the high mortality resulting from lymph node metastasis and distal metastasis. The review concludes that AI algorithms designed to diagnose tumor metastasis may be used as an adjunct by medical professionals with performance on par with healthcare professionals in terms of specificity and sensitivity [34]. The study calls for further comparisons of AI systems against healthcare professionals, additional measures of external validation, and rigorous reporting standards.

Artificial intelligence models, in conjunction with automated image recognition technology, have made large leaps and bounds in utility for the diagnosis of cardiovascular diseases and the estimation of prognosis and outcomes. With improvements in the quality of imaging, the diagnosis and assessment of coronary artery disease, and cardiac function, the way in which major adverse cardiac and cerebrovascular events (MACCE) are determined is being revolutionized. A narrative review detailing the advancements of AI in cardiovascular medicine details 24 and 14 studies using CT/MRI methodologies to study various aspects of cardiac function. These include current applications of AI in cardiac clinics such as diagnosis of myocardial infarction, prognosis of coronary artery disease, left ventricular myocardial analysis, plaque analysis, fractional flow reserve CT, coronary CT angiography and calcium scoring, perivascular adipose tissue, and the diagnosis and prognosis of cardiomyopathies (of all types-congenital, dilated, or hypertrophic) [35]. The study examines the performance of these systems across multiple cardiac indicators and estimates good accuracy, area under the curve (AUC), and correlation with manual reference [35]. The metric for estimating outcomes based on coronary artery calcium is a good predictor of cardiovascular mortality. A study determined this by analyzing risk factors and their correlation to coronary heart disease (CHD) in 6,814 intermediate-risk participants in a US-based large-volume multi-ethnic trial. Upon testing for outcomes such as angina, myocardial infarction, resuscitated cardiac arrest, or CHD death from CHD, statistically significant predictors were found to be coronary artery calcium, family history, high-sensitivity C-reactive protein (CRP), and ankle-brachial index [36].

In 2019, a study from the UK presented a new method to estimate cardiac risk, made possible by artificial intelligence. The authors of the study hypothesized that inflammation is the starting point for atherogenesis and resulting stenosis and that they could look to identify signatures of adverse coronary re-modeling (such as fibrotic degeneration or insertion of microvascular perivascular adipose tissue). This was accomplished by first taking biopsy samples from 167 patients undergoing cardiac surgery and subsequently isolating the sampled genes for fibrosis, vascularity, and inflammation. Then, the radiomic features of 101 patients with major MACCE within five years of receiving a coronary CT angiography were compared to 101 controls to identify the specific radiographic differences and fine-tune the AI model. This resultant fat radiomic profile was then applied to 1575 eligible participants in the Scottish Computed Tomography of the Heart (SCOT-HEART) trial, which found a significant improvement in risk stratification beyond existing gold standards such as coronary calcium score and coronary stenosis [37].

In emergencies, missing a fracture holds debilitating consequences for patients, delaying treatment and hampering recovery of function. Misdiagnosed or missed fractures account for a high percentage of reported diagnostic errors in certain emergency departments. A study in 2018 trained a deep learning algorithm with the notes of 18 seasoned subspecialized orthopedic surgeons on 135,409 radiographs. A group of emergency medicine clinicians were then asked to determine evidence of wrist fractures with and without the assistance of the previously developed deep learning model. The results show that the average clinician tested had a sensitivity of 80.8% unaided and 91.5% aided, with a specificity of 87.5% unaided and 93.9% aided. A reduction in the rate of misinterpretation was calculated at 47.0%. All values had appropriate 95% confidence interval values [38]. This is a statistically significant improvement in diagnostic accuracy, denoting the help that deep learning models can give clinicians. The authors of this study note that the current limitation of many computer-assisted detection systems of this nature is the underlying algorithm and its predefined parameters for textures or shapes in image detection. This leads to a high number of non-pathological regions being flagged overzealously [38].

One study out of Japan worked to develop a deep convolutional neural network (DCNN) to diagnose hip fractures in a cohort of 327 patients with proximal femur fractures. The comparative gold standard used was CT and MRI, graded by seven professional observers who evaluated 25 patients from the 327-patient cohort and 25 controls. The DCNN was trained on the remaining 302 patients, and subsequently tested against the trained readers. To compare the performance in detecting fractures of the two testing groups, this study utilized AUC. The seven trained observers had an AUC of 0.832, whereas the AUC of the DCNN was 0.905. When the readers utilized the DCNN output, the AUC was 0.876, a statistically significant (p <0.05) increase from readers alone without DCNN output [39]. Many remote hospitals and primary treatment centers may not have a radiologist available at all hours around the clock. Leveraging the help of AI to boost the sensitivity of fracture diagnosis in the emergency setting will be critical to improving this area of patient care. An algorithm created by researchers from Stanford in conjunction with a large trauma center located in Taiwan was found to have AUC values of 0.97 and 0.98 [40]. These values were obtained by comparing a single-center cohort of 4,235 pelvic X-rays (PXRs) against two validation cohorts from two hospitals on separate continents. This algorithm has the ability to upload PXRs from the bedside or a mobile phone and uses a convolutional neural network (CNN) to overlay heatmaps at suspected fracture locations [40]. Tools of this nature can be made readily accessible to supplement decision-making. Another study utilized a DCNN in the pursuit of reducing missed fracture diagnoses as well. This was accomplished by pretraining on 25,505 limb radiographs, and then retraining on 3,605 x-rays. The final developed visualization algorithm was purported to have 95.9% accuracy for the identification of lesions [41].

Due to the high risk of fracture in persons with low bone density, namely older adults, a routine dual x-ray absorptiometry (DEXA) scan is performed with great volume in the USA [42]. One study aimed to create a computer vision algorithm utilizing support vector machine learning to identify incidental findings on a DEXA scan. The goal of this algorithm was to eliminate the need for additional vertebral fracture assessment and to limit the burden on radiologists who oversee this high-volume procedure [43]. The authors of this study note that underlying differences among genders must be taken into account in the creation of this algorithm. The patient cohort used was one with a high prevalence of fractures, and as such, its results may not be entirely generalizable to the whole population [43]. A DCNN created by researchers from Taiwan was pretrained from ImageNet and retrained on 1,306 pelvic abdominal frontal radiographs. Of these 1,306 images, only 46.6% of the images with a vertebral fracture received the diagnosis of a vertebral fracture. The trained DCNN algorithm achieved 73.59% accuracy with an AUC of 0.72 in identifying vertebral fractures [44]. Numerous studies are emerging that detail the use of DCNN to accurately diagnose dangerous diagnoses with wide prevalence, though it is not clear yet how adequately applicable their results can be to overall populations. Researchers from Finland have created a more challenging test environment for an existing DCNN (DeepWrist) used for the detection of wrist fractures. The hypothesis presented in this study detailed subsets of cases of wrist fractures that eluded diagnosis without the clinical overseer requiring a CT. The coming of automated diagnostic assessments warrants a need to identify diagnostically challenging injuries [45].

A very familiar clinical setting for many clinicians is the suspicion of a scaphoid fracture in a simple fall. This injury presents serious consequences due to the scaphoid bones' unique blood supply, resulting in potential avascular necrosis in the carpal bone. A study in Turkey developed a CNN to analyze 390 anteroposterior wrist radiographs to diagnose scaphoid fractures. The diagnoses were confirmed by CT and compared to the opinions of ED physicians and two trained orthopedic specialists (experienced in hand surgery). The outcomes of the study were measured with AUC, sensitivity, specificity, F-score, and Youden index, and the CNN was found to have an AUC of 0.840. It ranked above the ED physician, and below the performance of the orthopedic specialists. The study concludes that in the absence of an experienced specialist, this tool would be a good adjunct in the diagnosis of scaphoid fractures [46]. To compare the utility of CNNs in scaphoid fracture detection, Langerhuizen et al. retrospectively collected 150 scaphoid fracture radiographs and 150 non-fracture radiographs with corresponding gold-standard CT/MRI for reference. A trained CNN was found to have an AUC of 0.77 (95% CI 0.66 to 0.85), with adding demographic data increasing a statistically insignificant amount to an AUC of 0.81 (95% CI 0.73 to 0.89). The CNN was also found to have numerous false positives which were correctly identified by five orthopedic surgeons, denoting the poor performance of CNNs when compared to trained specialists [47]. A systematic review conducted by Langerhuizen et al. examined 10 studies involving fracture detection and classification and reported the ranges for accuracy and AUC for the task/assessment. In concluding this review, the authors noted that more challenging diagnostic and therapeutic scenarios are necessary to test the neural networks in scenarios where the diagnosis is not so sure [48].

A single-center analysis from 2006 analyzed a cohort of 2,407 new patients with a total of 3,081 confirmed fractures in the ED across 18 months. They found 115 fractures in 108 patients who initially missed diagnosis, resulting in a 3.7% rate of missed diagnosis in fractures. Of these, 33% were attributed to radiologically imperceptible lesions [49]. With a growing burden on healthcare systems and a rise in clinician burnout [50], AI systems have become easy to see as the answer, with novel ideas for algorithms being theorized for a wide range of diseases. Smets et al. detail advances in machine learning for solutions in the management of osteoporosis, such as identifying new risk factors and associations and improving the current prediction models for fractures [51]. A meta-analysis of a pool of 1,574 images from nine trials was conducted to determine the reliability and accuracy of deep learning algorithms in orthopedic fracture detection. The results denote a high diagnostic accuracy with an AUC of 0.95, although the authors note that many included studies did not indicate a statistically significant improvement from orthopedists trained in the anatomical location of the fracture, only an improvement from general physicians and such [52]. Adams et al. conducted an experiment in 2019 where they trained two DCNNs (AlexNet and GoogLeNet) in the detection of neck-of-femur fractures to compare against medically naïve individuals given perceptual training. To adequately display a comparison, multiple datasets of 200, 320, and 640 images were used for training and validation, with an additional 160 images used as the final testing set. This study used multiple pre-processing and augmentation techniques. The accuracy of both groups’ performances was comparable, with the forthcoming conclusion being that the impressive results of DCNNs may be dwarfed by an hour of perceptual training by top-performing medically naïve individuals [53].

To increase adoption and application in the medical field, reporting standards with good external validation and comparisons with existing healthcare professionals are urgently needed [34]. Many studies comparing AI to the healthcare system of today are retrospective and contain varying standards of reference. Utilizing a verified diagnostic standard is of utmost urgency to show the authenticity and validity of future trials with AI. Without such a metric, it is difficult to quantify the diagnostic level of a specialist compared to AI. Due to the nascent nature of this field of medicine, studies are limited to relatively small sample sizes, often causing conclusions that are ungeneralizable to the population as a whole. These patient samples may appear random, but they are pre-designated diagnoses rather than stratified by patient demographics like geographical locations or ethnic groups. Studies utilizing gold-standard diagnostic methods for the DNN/CNN models have been observed to be, at times, limited in terms of diagnostic complications. Injuries or diseases requiring additional diagnostic tests or radiograph views from different angles are not accounted for sufficiently. This is related to the multi-organ burden of certain diseases, which will require further parameters to be added in future AI models. Larger datasets with more comprehensive patient information (physical exam, medical history, pre-treatment methods, etc.) can be included to refine the diagnostic algorithms. The DNN/CNN models can be compared to a neuron, with exponential learning capabilities based on the amount and quality of data they are trained on. This is also dependent on image processing quality. Insufficient data training will lead to an inaccurate AI. This can be considered both an advantage and a disadvantage of AI, as a large dataset will not over-encumber it. However, care must be taken to avoid bias in the provided dataset. This can be observed in datasets with a high percentage of outliers with a high certainty for diagnosis, i.e., radiographs from the ED. A skewed dataset with a hidden bias of this nature may result in a higher possibility of perceived diagnostic accuracy.

Socioeconomic challenges

Artificial intelligence has gained momentum in emergency medicine and critical care by providing predictive models and clinical decision support systems that match or exceed the results of experienced clinicians. Incorporating AI into healthcare, specifically in emergency medicine, has attracted notable interest because of its capacity to improve diagnostic precision, optimize processes, and enhance patient results. Despite the potential to improve patient care and outcomes, the acceptance and implementation of AI tools in this sector present several socioeconomic obstacles that must be carefully explored. The impact of socioeconomic factors on AI cannot be overlooked. Being data-driven, AI in healthcare reflects the socioeconomic disparities that can be biased against certain populations. For instance, people from lower economic classes may have less access to preventive or emergency care. Similarly, a lack of education, socioeconomic class, and race can all contribute to biased outcomes [54]. Artificial intelligence enhances and supports human intelligence rather than replacing it entirely. When developing AI systems for healthcare, it is crucial to preserve the essential elements of human interaction in medicine while also optimizing them to improve efficiency and effectiveness. The advancement of AI in healthcare requires a thorough and compassionate understanding of the intricate patient experience and pathways of care [55].

In research guided by user input, understanding its core issues is essential. By utilizing qualitative research, insights have been gained into the issue, causes, significance to stakeholders, reasons for neglect, and other factors. This extends to grasping healthcare operational processes, limitations, and factors aiding or hindering AI integration in clinical contexts. Once understanding deepens, the next step involves identifying AI-suitable issues. Evaluating available datasets for AI model creation and assessment is vital. Integrating algorithms into existing workflows is crucial for aligned operations and their adoption. The goal is to find relevant solutions for end-user challenges. Focusing on experiments is key. Inputs taken from all stakeholders allow rapid experiential learning, which in turn helps us refine AI tools. This clarifies AI's purpose, potential users, and risks like data privacy, security, and fairness. Responsible AI deployment in healthcare mandates addressing these aspects [55].

Economic accessibility

To successfully incorporate and utilize AI solutions in the healthcare sector, particularly in highly stressful environments such as emergency medicine and critical care departments, economic feasibility must be examined. The United States is the leader in providing advanced medical training, conducting research, and making technological advancements, especially in medicine. Studies show that they also have a higher expenditure, a quarter of which is considered wasteful and potentially avoidable. However, despite the high costs, when compared to other top-ranking countries such as Canada, Japan, Germany, the United Kingdom, Australia, France, the Netherlands, Switzerland, Denmark, and Sweden, it ranks low when it comes to healthcare outcomes and public services [56].

Supervision of AI and potential errors

Technology holds advantageous implications for the healthcare domain, especially concerning diagnosis and treatment procedures. With the capability to swiftly access real-time patient data through simple interactions with a screen, technology is currently facilitating rapid care administration. This advancement is poised to effectively manage urgent medical situations and subsequently mitigate potential casualties [56]. Artificial intelligence-driven devices like advanced CT scans, MRIs, and ultrasounds exhibit heightened accuracy in simple tasks, thereby reducing errors and costs and enabling early intervention. Artificial intelligence achieves 99% accuracy and outpaces humans in mammogram evaluation, expediting breast cancer diagnosis and cost efficiency. Utilizing data effectively empowers better decision-making across various sectors, including healthcare. In healthcare, vast data feeds AI algorithms for pattern-based analysis, enhancing timely decision-making through improved time analysis [56]. Artificial intelligence advances clinical decision-making and personalized healthcare, swiftly enhancing outcomes and reducing expenses linked to post-treatment complications, a significant cost factor in global healthcare systems [56]. Machine learning applications have dominated the field of medical imaging, including diabetes, cardiovascular disease, liver, thyroid, ovarian, and prostate cancers, as well as risk characterization using coronary and vascular screening using carotid angiography. Artificial intelligence will be effective for forecasting diagnosis and risk stratification for various diseases with good accuracy, a lower cost, and a shorter diagnosis time [56].

Artificial intelligence is pivotal in managing complex diseases like cancer. Integrating omics data links biomarkers to biological pathways. Artificial intelligence identifies cancer subtypes and potential therapeutic radiogenomics, predicting disease prognosis and treatment response. Radiogenomics selects patients based on genetics, diverging from traditional criteria. Radiogenomics' growth exploits radiomics, genetics, and clinical data with big datasets and advanced machine learning, creating new algorithms. Personalized treatment through radiogenomics relies on reliable predictive tools. For instance, Transparent Reporting of a Multivariable Prediction Model for Individual Prognosis or Diagnosis (TRIPOD) models individual outcomes. Advanced radiogenomics requires addressing gaps in radiobiological knowledge. Merging imperfect data with existing knowledge yields novel insights [56]. Nevertheless, the potential consequences of errors committed by AI systems can be disastrous, depending on the context of their application. In the medical sector, the well-being of patients hinges on these determinations, while an error in an unmanned vehicle's vision system might lead to an accident. To address these issues, the implementation of explainable AI (XAI) comes into play [56].

Overfitting

There's a persistent concern that AI could lead to job losses in healthcare, causing skepticism and resistance to AI-based initiatives. However, this view often stems from a misunderstanding of AI's diverse forms. Integration of AI won't necessarily render jobs obsolete, but it will require their adaptation. Healthcare processes are inherently complex and unpredictable, with a human element that algorithms can't fully replicate. Biases in data collection for model development could also lead to skewed results. Biases can occur due to data collection. When the algorithm grasps insignificant connections between patient characteristics and results, it encounters overfitting. This occurs when an excess of variables impacts outcomes, causing the algorithm to make erroneous forecasts. Consequently, while the algorithm might perform well during training, its predictions for future events can be unreliable [57].

Acceptability of AI

Then arises the challenge of healthcare professionals accepting the use of AI on the ground. Various authors have highlighted barriers and challenges in the implementation of EHRs and learning health systems (LHS). These barriers encompass financial burdens, legal complexities, data standardization, interoperability issues, and organizational cultural factors. Clinical users and patients often express reservations about computing systems, fearing disruptions to patient care workflows, time management, and training needs. Data accuracy concerns, potential impacts on patient privacy, and unresolved data security issues are also significant barriers. Budget overruns and overpromised technology further contribute to negative perceptions of EHR and LHS, with these barriers consistently identified for both systems [58].

For the effective adoption of medical AI by healthcare workers, service developers should prioritize performance and effort expectations. Understanding healthcare workers' needs, enhancing AI-related research and development, ensuring accurate information and professional functions, and improving efficiency and service quality are crucial. User-centered service providers should focus on user experience, ease of operation, and interface friendliness to facilitate adoption. Hospital administrators play a role by fostering trust in AI through supportive management strategies. Encouraging adoption, providing training, linking performance with incentives, and showing organizational support can enhance AI acceptance among healthcare workers. Government involvement is key to promoting AI-assisted healthcare. Amplifying publicity and social influence, along with mandatory adoption, can accelerate technology acceptance. Positive social attitudes towards AI, built through effective promotion and word-of-mouth, can significantly influence healthcare workers' adoption intentions [59].

It is also important for patients to accept the use of AI to benefit their care. Patients can benefit from AI tools in healthcare, but their opinions matter for widespread use. Patients must be assured that AI won't harm them and can help their health. Before using AI in regular care, concerns and risks need attention. Technical, ethical, and regulatory worries affect how risky AI seems. Technical issues, like how well AI works, matter the most. People decide if AI is good based on its value compared to the risks. If concerns aren't addressed, AI might not be trusted and could be seen as a threat to health. Makers of AI should show benefits and address worries. Rules should define roles and responsibilities for safe AI use by healthcare pros, developers, and users [60]. There is growing evidence that AI-predictive models can perform on par with seasoned clinicians in emergency medicine and critical care. This means AI can be used to identify and even treat patients with certain conditions, in turn giving providers the support of AI to act in a timely and efficient manner [61,62]. Although the use of AI in emergency medicine has the potential to transform healthcare, the socioeconomic [55] hurdles it poses during implementation should not be overlooked. Recognizing and tackling these challenges is crucial to ensuring the ethical, fair, and lasting integration of AI technologies. As healthcare advances, collaboration among researchers, providers, policymakers, and AI creators will be key to effectively navigating these challenges.

Racial, social, and economic bias

There is a growing concern about the various biases that are associated with AI in healthcare. The literature, policy frameworks, and legal decisions regarding liability related to AI technologies are largely based on the EU and the USA, where these technologies are actively used. It's uncertain whether these approaches will be adopted in lower-middle-income countries (LMICs) or if different strategies will emerge. Liability rules are essential for safety and accountability, acting as the primary defense against errors from machine-learning technologies. Many LMICs lack the regulatory capacity to assess new technologies and their potential benefits. Concerns about AI technologies operating as intended are amplified by the lack of quality data for training algorithms and the potential for contextual bias [63]. Artificial intelligence algorithms are trained on historical and demographic data that can perpetuate racial bias in healthcare. This means that based on the data, there is a high probability that AI can conclude that certain conditions are characteristic of certain groups of people, even if this is not entirely true. This leads to biased health outcomes, and discrimination can affect such a group of people in terms of insurance coverage [64].

Growing worries involve algorithms perpetuating racial and gender disparities, possibly through the data used to train them or the individuals constructing them. Empirical evidence supports these concerns, revealing biases in areas like job searches, facial recognition, and natural language processing [64]. For example, if datasets have fewer minorities due to biased data collection, AI's predictions might not be accurate. Ways to address this bias include using diverse training sets. Some AI models can even handle bias independently, like a neural network that lessens the impact of unclear elements [57]. A concept called bias attributable to label choice is valuable in understanding algorithmic bias, as labels often reflect existing inequalities. To address this, changing the labels used for training requires a deep domain understanding, relevant data extraction, and experimentation. While challenging, similar practices are seen in private companies developing predictive labels. Despite the challenges in sectors like health, criminal justice, and employment, investing in research for improved labels is crucial. The choice of labels significantly impacts predictive quality and bias, allowing us to harness algorithmic predictions while minimizing risks [64].

Artificial intelligence systems rely heavily on enormous volumes of patient information to function adequately. How objective AI algorithms are depends on the data that they are trained on. An unfair disadvantage may be caused by biased statistics for specific patient populations. Researchers Obermeyer et al. (2019) demonstrated the existence of racial bias in a widely used AI system for the healthcare industry that came across due to biased data input. To resolve this ethical issue, emergency medical practitioners must carefully examine the data used to train machine learning algorithms, actively uncover and minimize biases, and routinely check for potential discriminatory implications [64].

Deficient legal framework

There is a lack of legal framework to address issues that arise with AI. Whenever medical procedures, tools, or services are used, there are risks involved. Patients expect a certain level of care and quality. Laws often mention the care patients should reasonably expect. However, it's challenging to decide who is responsible and who owes care when issues arise from using the EHR and LHS. Laws usually handle cases when doctors fail in their care duties, but they don't fully cover issues related to various eHealth products like EHRs, diagnostic devices, or medical apps [58,63]. The use of AI in emergency medicine and critical care could pose an insurance risk, and it is important to address these challenges to ensure equitable insurance coverage for all patients. This is because AI can accurately detect the patient's outcome and risks with their condition; this can lead to insurance companies withdrawing coverage for high-risk patients or patients with low socioeconomic status [65]. There is a threat of job replacement in the healthcare setting with the use of AI. However, it should be noted that AI can more likely complement healthcare workers than replace them since the human touch is an essential part of healthcare. Artificial intelligence-powered tools can be used to supplement the skills and knowledge of physicians and other healthcare workers, which can ultimately lead to better outcomes [54]. In conclusion, AI in primary care and critical care has the potential to be beneficial, but the associated challenges and risks must be addressed to improve clinical outcomes for patients regardless of their background.

In the context of emergency medicine, AI has the potential to revolutionize patient care, optimize resource allocation, and improve clinical decision-making. However, the implementation of AI in emergency medicine also brings forth several ethical challenges that demand careful consideration. As the use of AI in emergency medicine is evolving, it is paramount to secure the safety, privacy, and moral use of such technology. However, the application of AI in medicine raises several concerns [16]. Artificial intelligence is capable of learning by recognizing patterns through the analysis of large amounts of data. To “teach” AI, an enormous amount of data input is required to train the algorithm. The desire of developers to gather as much data as they can must be weighed against patients' right to privacy [57,66].

Data privacy and policies

Security breaches and unlicensed access to personal health records are risks that are always present when AI algorithms process confidential medical data. Data integrity and privacy pose significant ethical issues. To adequately safeguard patient data and hold data controllers accountable, information laws such as the GDPR process personal data through a union-based data processor or controller and provide individuals with increased privacy and control of their personal information [16]. Notably, in the deal made between Google DeepMind and the Royal Free London NHS Foundation Trust, the data of millions of patients were annexed without explicit consent or prior information from the patients, despite existing privacy policies and regulations. This is a stark example of the lack of strict laws and principles that are needed to keep up with the rapidly developing field of AI [67,68].

Data errors

It is important to note that most models are trained on retrospective data. Retrospective data is subject to potential errors in data entry. Information could be missing, inaccurate, or altered. This can lead to associations between variables and outcomes even where no such link exists [69]. In addition, algorithmic errors have to be taken into account. While automated diagnosis can aid decision-making, an automation bias can lead to an incorrect diagnosis [70]. One of the highest-risk environments in medicine is the emergency department, where a variety of elements, including time restraints, pressure, rapid job transitioning, and elevated cognitive demand, can have a detrimental effect on decision-making. Artificial intelligence may offer innovative solutions for assisting doctors in dealing with the issues of a diminishing workforce and expanding healthcare demand [71].

The black box dilemma

“Explainability" is a phrase used to describe the black-box dilemma. The black box of AI is a problem where the methods used to interpret the data are not fully understood. In particular, predictive AI systems analyze vast volumes of data to identify undiscovered patterns and offer solutions. Accepting the results of such evaluations is challenging because we are often unable to understand why the programs arrived at their conclusions. Explainability is the capacity of an AI system to be understood by humans through an external, simplified representation. The XAI movement has grown as a result of such factors. It is based on the idea that AI algorithms currently carry out important jobs, making them 'white boxes' that are transparent to both their designers and end users (or anybody else who could be impacted by the algorithm's decision). Although humans do not understand the pattern or stages involved in the algorithm's determination, it employs a computer to arrive at a conclusion in a few seconds. This calls into question the openness of ML algorithms [72,73].

Hallucinations

Artificial intelligence models have another challenging issue at hand, referred to as "AI hallucinations,” wherein large language models like ChatGPT or image reconstruction models can integrate non-existent or “imaginary” artifacts into their outputs [74, 75]. It can be dangerous to introduce false structures into our data, and the use of tools such as a hallucination map enables the examination of flaws in a reconstructed object's data component [76].

Is AI trustworthy?

The issues stated above can spark the question of whether or not we can rely on AL at all. A framework written by the high-level expert group on AI (AI HLEG) outlines the three components of trustworthy AI: being lawful, robust, and ethical. The following (Table 1) requirements, if met, can help ensure AI’s trustworthiness.

**Table 1:** Requirements to ensure AI's trustworthiness AI: artificial intelligence; AI HLEG: high-level expert group on artificial intelligence Reproduced under the terms of the Creative Commons attributions license [77, 78].

Requirements to ensure AI's trustworthiness
Supervision by humans
Robust technology with resilience to attack and security, a fallback plan
General safety, accuracy, reliability, and reproducibility
Respect for privacy, quality, and integrity of data, and access to data
Transparency, including traceability, explainability, and communication
Diversity, non-discrimination, and fairness, including the avoidance of unfair bias, accessibility and universal design, and stakeholder participation
Societal and environmental well-being, including sustainability and environmental friendliness, social impact, society, and democracy
Accountability, including auditability, minimization and reporting of negative impacts, trade-offs, and redress

It is critical that AI is incorporated into the healthcare sector correctly and wisely, as it can be a win-lose situation. Thus, any advantage would definitely be a feather in the cap. Studying the behavior of the users, understanding the software, and trusting the process are the key factors that can determine the overall performance of the AI/ML tools. Examining the tools of AI by regulatory bodies in clinical research across phases would involve a multidisciplinary approach with expertise in healthcare and sociology, information technology, computers, etc. AI and ML have the ability to modify healthcare through real-time, day-to-day-generated insights. The FDA in the U.S. considered AI/ML as part of the "Software as a Medical Device" (SaMD) action plan from the Center for Devices and Radiological Health’s Digital Health Center of Excellence [79,80]. The International Medical Device Regulators Forum (IMDRF) defined SaMD as intended to be used for any medical purposes without having to be part of a medical device [80]. Under the Federal Food, Drug, and Cosmetic Act (FD&C Act), medical purposes are considered those purposes that are intended to prevent, diagnose, treat, cure, and mitigate disease conditions [80]. This ability of AI to receive feedback to improve performance and adapt makes AI/ML unique and is considered a SaMD [79]. The submission type and data requirements are based on the risk of the SaMD (510(k) notification, de novo, or premarket approval application (PMA) pathway) [80].

The FDA has been in the process of composing AI/ML clinical use-approved specific guidelines [79,80]. The idea of the regulatory bodies is to set the stage for the safe transition of AI to clinics [81]. The challenges that regulatory bodies might face with AI use in clinical practice are to assess continued transparency, predictability, accountability, repeatability, etc. [82-85]. It is paramount that these factors are assessed, as there will be numerous promising AI applications in the near future. In the past, as the benefits and risks of AI have yet to be adequately established in clinical practice, foundational studies were criticized by policymakers [81]. The FDA's regulation for AI/ML algorithms explains how to go about each step of evaluation rather than specific directions [79]. The evaluation of each phase for AI considerations in healthcare has been specified [86]. The FDA reviews the AI/ML applications through appropriate pathways: premarket clearance (510(k)), de novo classification, or premarket approval, algorithm change protocol (evaluating and monitoring performance from pre- to post-market stage), etc., to ensure patient safety. Modifications are reviewed by the FDA depending on the significance or risk posed. Evaluation practices include IMDRF risk categorization principles, a benefit-risk framework, risk management principles, and the total product lifecycle approach (organization-based) [80].

The FDA wants to deliver effective, safe AI/ML software that can improve the quality of patients’ care. Artificial intelligence/machine learning-based SaMDs are intended to be categorized into four categories, from lowest risk (I) to highest risk (IV), to associate the risk associated with device use with the clinical situation [80]. Until now, the FDA has approved or cleared several AI/ML-based SaMDs that involved only 'locked’ (each time, same result) algorithms. As only some AI/ML software is locked (as algorithms tend to adapt after learning from real-world experience over time), the regulations might not be designed for adaptive AI/ML technologies that can improve healthcare for patients in real time [80]. A total product lifecycle (TPLC) regulatory approach for these kinds of adaptive software is required [80]. These ML algorithms that continually evolve are often known as “adaptive” or “continuously learning” algorithms, as they don’t actually need a manual modification to incorporate learning or updates required for the software. Whereas adaptive algorithms can learn from new user data that are presented to them through real-world use, the regulatory framework for modified algorithms from real-world adaptation and learning is still being explored [80]. Both diagnostic and therapeutic AI interventions might face specific hurdles (potential bias related to AI/ML, etc.) that are inadequately addressed by the Consolidated Standards of Reporting Trials (CONSORT) and Standard Protocol Items: Recommendations for Interventional Trials (SPIRIT) [80,87,88]. These are considered the first international standards for clinical trials of AI/ML, and they have been used to ensure transparency, rigor, and reproducibility [80,87,88]. Machine learning prediction model studies should be specific; they need to assist with robust scientific evaluation and adopt established guidelines for reporting and the development of TRIPOD [80,87].

It would be more helpful if individual AI models were focused, evaluated, and targeted instead of using generalized models. Specific data streams can be considered at a particular touchpoint along the pathway of patient care. Possible touch points can be considered: screening, relative risk assessments, diagnosis, initial management, prognosis, responses, and follow-up [89]. To provide and see a more precise clinically measurable benefit, institutions and clinicians should use and focus on narrowed AI/ML care, performing narrow-focused tasks, and using a single data stream for specific touchpoints along the disease course of the patients, so that at the end, results can be accumulated to find a more profound benefit [89]. Because there has been a continuous gap between the clinical influence of AI/ML and its performance [89], not only the kind of data used to train the AI/ML algorithm might be defective, but also aggregation, curation, development of transparency, trust, reliability, interpretability, and reproducibility of the data used for AI models could have been a huge concern [90-92]. Under-represented data used to train the AI/ML algorithm can be another concern while predicting the performance of edge cases [93]. The ideal gold standard design for the AI/ML algorithm would be the one that randomizes patients to the AI/ML algorithm involving intervention and then directly compares the results with the target population and the clinical endpoints. Table 2 shows considerations for incorporation at each phase of AI/ML algorithms or software to achieve a standardized approach.

**Table 2:** Consideration for incorporations at each phase of AI/ML algorithms or software to achieve a standardized approach. Reproduced under the terms of the Creative Commons attribution license [86]. AI/ML: artificial intelligence/machine learning

Phase	Objectives	Methods/Study designs	Considerations
Phase 0	1) Quality data; 2) Focus on target population	1) Using descriptive analysis for algorithm testing and quality control	1) Change of quality and data; 2) Separate testing for acceptable accuracy standards depending on clinical consequences
Phase 1	1) Identifying user needs and workflow; 2) Determining useful functionalities and design options; 3) Understanding socio-cultural backgrounds for effective clinical decision-making.	1) Using observational workflow analysis in ethnographic research	1) This phase should precede prototype development; 2)Results should not be generalized across varied settings.
Phase 2	1) Controlled experiment for effect-size estimate; 2) Using crossover designs; 3) Usability testing	1) Simulation, A/B testing; 2) Expert reviews to test usability	1) Controlled experiment needed; 2) A carryover effect can invalidate crossover study results; 3) Researchers should consider the full context of AI/ML software use (workflow and clinicians’ mindset).
Phase 3	1) Implementation of blinding, if appropriate; evaluate cluster effect	1) Using randomized clinical trials, cluster trials, step-wedge trials, and pre-post comparison trials.	1) Robust study designs are required; 2) Blinding is required to avoid contamination; 3) Large enough cluster size should be considered.
Phase 4	1) Evaluate in real-world, multi-center settings and assess the time-adjusted effects; 2) Pre-implementation data controls used to evaluate post-implementation data for the same cohort.	1) Using prospective observational cohort studies and retrospective cohort studies; 2) Feedback and surveillance with data collection	1) All site implementation; 2) More resource intensive; 3) Control for time invariant factors; 4) May be subject to bias even more at this phase; 4) Confounding bias; 5) User feedback system should be built in to receive data continuously and to update AI/ML software.

It is of utmost importance that AI/ML algorithms or software are safely and effectively incorporated, tested; approved, commissioned, continuously and systematically reported in real-time to the top standards using multidisciplinary expertise using ethically relevant approaches and resources so that AI/ML interventions can be built-in for the long term and embedded in daily standard clinical care without compromising on patients’s safety. Even though AI/ML may be rigorously evaluated and regulated, AI/ML software or algorithms tend to be potentially prone to biases (diagnostics and therapeutics) or technical errors or limitations. Long-term prospective studies using electronic records of patients' health or using a database that involves health insurance claims; understanding the user needs and satisfaction through continuous feedback, to improve the interface once the AI/ML products are on the market; continuous surveillance; prompt reporting of any unexpected or unexplained potential adverse events; pre- and post-marketing, etc., should all be factors to be focused on while composing a regulatory framework for both the locked and adaptive AI/ML software or algorithm so that patients and healthcare systems receive the benefits of these relatively new, promised advancements in medicine for the longer term. However, concepts like utility, usability, clinical validation, etc., should not be overlooked as they can transition AI/ML algorithms or software from research and development to real-world patient care.

In the domain of healthcare, the assimilation of AI emerges as a potent catalyst, poised to uplift patient care, optimize resource distribution, and streamline administrative functions. To harness AI's potential fully, healthcare policymakers need to actively engage with ethical, regulatory, and practical complexities. This article extends guidance to these policymakers, providing insight into pivotal aspects, potential benefits, and essential precautions when incorporating AI into healthcare frameworks. The advice shared here originates from well-established literature and expert perspectives, fostering informed policy decisions and advocating for the thoughtful integration of AI into healthcare contexts. In contemporary times, the substantial growth of AI has permeated diverse sectors, including the realm of healthcare. This article underscores AI's role within healthcare and seeks to equip policymakers with grounded recommendations for navigating this dynamic landscape. The advantages of AI in healthcare span diverse dimensions, encompassing elevated diagnostic accuracy, personalized treatment routes, and enhanced administrative efficiency. Applications powered by AI encompass tasks like analyzing medical images, uncovering novel pharmaceutical compounds, facilitating virtual healthcare aids, and enabling predictive analytics.

The ascent of AI introduces ethical dilemmas pertaining to privacy, security, bias, and accountability. Policymakers bear the responsibility of collaborating with stakeholders to establish ethical benchmarks, enforce data protection protocols, and ensure the responsibility and transparency of AI algorithms [94]. Creating a comprehensive regulatory structure becomes pivotal in governing AI-driven healthcare applications. Policymakers need to collaborate with relevant entities to formulate standards that guarantee safety, effectiveness, and adherence to medical regulations. The integration of AI relies on patient data, necessitating that policymakers establish robust privacy and security measures. This involves adhering to the principles of informed consent and de-identification to effectively safeguard patients' sensitive data from unauthorized access and breaches [95]. To optimize AI's benefits, healthcare policymakers should invest in training healthcare personnel to adeptly engage with AI technologies. This encompasses addressing potential concerns related to job displacement and upscaling the workforce to fit emerging roles.

Realizing the complete potential of AI hinges on the seamless exchange and accessibility of healthcare data across institutions. Policymakers should strive for standardized data formats and agreements facilitating data sharing, thereby bolstering AI-driven innovations. Artificial intelligence algorithms can unintentionally perpetuate biases present in training data, potentially leading to disparate outcomes among specific patient groups [64]. Healthcare policymakers are tasked with prioritizing the creation of strategies to mitigate bias and ensure a fair and comprehensive application of AI. It's imperative for policymakers to mandate that AI models deployed in healthcare offer clear and interpretable explanations for their decisions. This commitment to transparency in AI algorithms enhances patient trust and facilitates stronger collaboration between medical professionals and AI systems. Healthcare policymakers should institute mechanisms for the ongoing evaluation and oversight of AI applications. Regular assessments can identify potential challenges, foster ongoing improvement, and cultivate public confidence in AI-driven healthcare solutions [96]. Cultivating an environment of collaboration involving researchers, medical practitioners, industry stakeholders, and policymakers is pivotal for advancing AI in healthcare. Encouraging multidisciplinary collaboration expedites the development and adoption of innovative AI solutions. Embracing AI within healthcare necessitates prudent planning, robust regulations, and unwavering dedication to ethical considerations. By implementing the insights discussed in this article, healthcare policymakers can ensure the responsible integration of AI, leading to improved patient outcomes, heightened efficiency, and an equitable healthcare landscape.

## Conclusions

Artificial intelligence is not free from its imperfections. This article has discussed concerns such as data privacy, the cost of AI, substandard regulation, inadequate data, a lack of standardization, and poor acceptance by patients and doctors. To mitigate these issues, we require policies and regulations to encourage the use of AI while protecting human rights that are in keeping with the principles of medical ethics (including autonomy, beneficence, non-maleficence, and justice in all aspects of healthcare), regulatory bodies, and the laws of various countries. Adequate training in AI requires using sizable and unbiased datasets while safeguarding protected health information. Responsible integration of AI in healthcare, particularly in emergency medicine and critical care, while ensuring cautious and supervised use of AI by physicians and healthcare workers to mitigate potentially fatal errors and ensure accurate AI predictability. Multidisciplinary expertise to develop AI that is transparent, trustworthy, clinically valid, acceptable, accessible, and affordable, long-term studies with validated and standardized protocols that can be generalized, and continuous surveillance of AI for unintended errors and adverse events are required. There is an overestimation of the convenience and limitations of AI in healthcare. It is not a requirement that all physicians know how AI works; however, learning to utilize AI just as any other tool with precaution can prove to be beneficial. We must temper our expectations and back them up with reliable research.
